# Genome-wide identification, structural characterization and gene expression analysis of the WRKY transcription factor family in pea (*Pisum sativum L.*)

**DOI:** 10.1186/s12870-024-04774-6

**Published:** 2024-02-16

**Authors:** Ruiqi Xiong, Zhonghua Peng, Hui Zhou, Guoxing Xue, Ailing He, Xin Yao, Wenfeng Weng, Weijiao Wu, Chao Ma, Qing Bai, Jingjun Ruan

**Affiliations:** 1 College of Agriculture, Guizhou University, Huaxi District, Guiyang, Guizhou Province 550025 P R China; 2 Sichuan Province Seed Station, Chengdu, Sichuan 610041 China

**Keywords:** *Pisum sativum L.*, *WRKY* gene family, Genome-wide identification, Transcription factor, Hormone treatment

## Abstract

**Background:**

The *WRKY* gene family is one of the largest families of transcription factors in higher plants, and WRKY transcription factors play important roles in plant growth and development as well as in response to abiotic stresses; however, the *WRKY* gene family in pea has not been systematically reported.

**Results:**

In this study, 89 pea *WRKY* genes were identified and named according to the random distribution of *PsWRKY* genes on seven chromosomes. The gene family was found to have nine pairs of tandem duplicates and 19 pairs of segment duplicates. Phylogenetic analyses of the PsWRKY and 60 Arabidopsis WRKY proteins were performed to determine their homology, and the PsWRKYs were classified into seven subfamilies. Analysis of the physicochemical properties, motif composition, and gene structure of pea WRKYs revealed significant differences in the physicochemical properties within the PsWRKY family; however, their gene structure and protein-conserved motifs were highly conserved among the subfamilies. To further investigate the evolutionary relationships of the PsWRKY family, we constructed comparative syntenic maps of pea with representative monocotyledonous and dicotyledonous plants and found that it was most recently homologous to the dicotyledonous *WRKY* gene families. Cis-acting element analysis of *PsWRKY* genes revealed that this gene family can respond to hormones, such as abscisic acid (ABA), indole-3-acetic acid (IAA), gibberellin (GA), methyl jasmonate (MeJA), and salicylic acid (SA). Further analysis of the expression of 14 *PsWRKY* genes from different subfamilies in different tissues and fruit developmental stages, as well as under five different hormone treatments, revealed differences in their expression patterns in the different tissues and fruit developmental stages, as well as under hormone treatments, suggesting that *PsWRKY* genes may have different physiological functions and respond to hormones.

**Conclusions:**

In this study, we systematically identified *WRKY* genes in pea for the first time and further investigated their physicochemical properties, evolution, and expression patterns, providing a theoretical basis for future studies on the functional characterization of pea *WRKY* genes during plant growth and development.

**Supplementary Information:**

The online version contains supplementary material available at 10.1186/s12870-024-04774-6.

## Background

Transcription factors (TFs), also known as trans-acting factors, are a class of DNA-binding proteins that regulate gene expression and transcription rates by recognizing specific sequences of target genes and binding to specific cis-promoter elements [1]. TFs play important roles in regulating plant growth, morphogenesis, cell cycle, and adaptation to the environment [2–5]. TF genes (*bHLH*, *WRKY*, *MYB*, *bZIP*, and other TF family members) account for a large proportion of the plant genome, and their target genes are widely involved in physiological processes such as plant development and stress responses [6, 7].

The WRKY TF family is one of the largest TF families in higher plants, and WRKY TFs are a class of DNA-binding proteins that are found primarily in plants. The DNA-binding domain of WRKY TFs called the WRKY structural domain, is 60 amino acids long and characterized by a zinc finger motif and a highly conserved WRKYGQK sequence at the N-terminus [8, 9]. According to the number of WRKY structural domains and the type of zinc finger motifs, WRKY proteins can be classified into three classes: those with two WRKY structural domains belong to class I, and those with only one WRKY structural domain belong to classes II and III. Members of classes I and II have C2-H2 (C-X4-5-C-X22-23-H-X1-H) zinc-finger-like motifs, whereas class III WRKY proteins contain C2-HC (C-X7-C-X23-H-X-C) zinc-finger-like motifs, where X can be any amino acid [10]. Whole-genome sequencing for some plant species (especially model plants) has revealed more *WRKY* genes, for example, 197, 97, 74, and 109 WRKY superfamily members in soybean (*Glycine max* (L.) Merr.), rice (*Oryza sativa L.*), Arabidopsis (*Arabidopsis thaliana* (L.) Heynh.), respectively [11–13].

WRKY TFs are involved in a variety of plant processes, including growth, development, and stress signaling, through self-regulation and cross-regulation with different genes and TFs [14], are one of the most characterized classes of plant defenses, and have been at the forefront of research on plant defense responses. WRKY proteins were found to play a key role in plant defense against biotic stresses, including bacterial, fungal, and viral disease [15–17]. For example, *GmWRKY31* may act as a positive regulator in the activation of *GmSAGT1* gene expression in response to *Peronospora manshurica* infection [18]. Overexpression of *GmWRKY136*, *53*, and *86* increases soybean resistance to soybean cyst nematodes [19]. And *TaWRKY49*, *50*, *52*, *55*, *57*, and *62* may also play a role in leaf rust-induced biotic stresses [20]. In addition, WRKY TF is induced by abiotic stresses and is involved in regulating plant tolerance to abiotic stresses [21]. For example, *VvWRKY11* in grapes is involved in the response to dehydration stress [22], and plants overexpressing *AtWRKY39* exhibit enhanced heat tolerance [23]. WRKY TFs also play an important role in drought and salt tolerance in cotton (*Gossypium* spp) [24]. WRKY proteins are also key parts of certain signaling processes mediated by phytohormones such as SA, JA, ABA, and MeJA [25–27]. For example, *AtWRKY39* can positively regulate the cooperation between SA- and JA-activated signaling pathways to mediate the response to heat stress [28], and the data suggested that multiple sweet potato *IbWRKYs* might play significant roles in hormone signal transduction [29].

Pea (*Pisum sativum L.*) is a self-pollinating, cool-season legume, one of the most important and valuable legume crops worldwide. It is widely cultivated in temperate regions and possesses a wide range of qualities, such as high digestibility and palatability, high nutritive value, availability, and relatively low cost [30, 31]. Pea seeds are rich in protein and fiber, and are an excellent source of mineral nutrients but low in cholesterol and antioxidants, they can be eaten as a vegetable (green pods), dried peas, or a green manure crop that will also help improve soil fertility by fixing atmospheric nitrogen [32–34], and consumption of legumes, including peas, can help reduce the risk of cancer and cardiovascular disease, and the inclusion of peas in the diet regulates blood glucose and insulin levels, which helps control diabetes [35, 36]. Peas are highly susceptible to abiotic stresses, such as drought, heat, humidity, and cold. Globally, drought and high-temperature stresses during flowering are the major abiotic stresses and elevated temperatures can lead to flower or fruit drop, seed abortion, and seed size abnormalities [37]. Peas are beneficial to health and are widely consumed; thus, they have great economic and research value, and the identification of their functional genes is of great importance.

In 2022, the latest pea genome sequence was released [38], allowing us to better study its evolution, development, and gene function. The *WRKY* gene family has been identified in tomatoes, Phragmites, Arabidopsis, rice, and other crops, but not in pea. In this study, we identified Pea *WRKY* family members at the genomic level and analyzed their physicochemical properties, chromosomal distribution, cis-acting elements, gene structures, conserved motifs, and phylogenetic trees. Additionally, the tissue-specific and fruit developmental expression patterns of the major members of the PsWRKY family during the irrigation stage, as well as their gene expression levels under different hormone treatments, were analyzed to enable further understanding of their biological functions and evolutionary relationships.

## Results

### Identification of *WRKY* genes in Pea

A total of 89 members of the *WRKY* gene family in peas were identified by BLAST with *A. thaliana* and *O. sativa*, named *PsWRKY1*–*PsWRKY89* based on their chromosomal locations, and to analyze the 89 PsWRKY proteins for molecular weight (MW), theoretical isoelectric point (pI), subcellular localization, and other physicochemical properties (Table S1).

Of the 89 PsWRKY proteins, PsWRKY23 was the largest with 710 amino acids, and the smallest was PsWRKY47 with 154 amino acids, and the molecular weight results indicate that PsWRKY23 was the largest (79.12 kD), and PsWRKY47 the smallest (17.68 kD). The pI ranged from 4.94 (PsWRKY87) to 9.97 (PsWRKY35), with the majority of pI < 7, suggesting that this gene family is biased towards acidic amino acids. Predictive subcellular localization analysis from WoLF PSORT showed that 82/89 *PsWRKYs* were located in the nucleus, 2/89 in the cytoplasm, 1/89 in the mitochondria, 1/89 in the plasma membrane, 1/89 in the chloroplast, 1/89 in the Golgi, and 1/89 in the peroxisome, and the results of predictive subcellular localization analysis from Cell-PLoc were similar in 78%, but there are differences in the results of several gene analyses (*PsWRKY7*, *PsWRKY13*, *PsWRKY18*, *PsWRKY26*, *PsWRKY27*, *PsWRKY30*, *PsWRKY43*, *PsWRKY48*, *PsWRKY54*, *PsWRKY58*, *PsWRKY60*, *PsWRKY63*, *PsWRKY65*, *PsWRKY67*, *PsWRKY68*, *PsWRKY80*, *PsWRKY85*, *PsWRKY86*), and the specific results will be determined based on the experimental results.

### Phylogenetic analysis, classification, and multiple sequence alignment of PsWRKY proteins

**Fig. 1:**
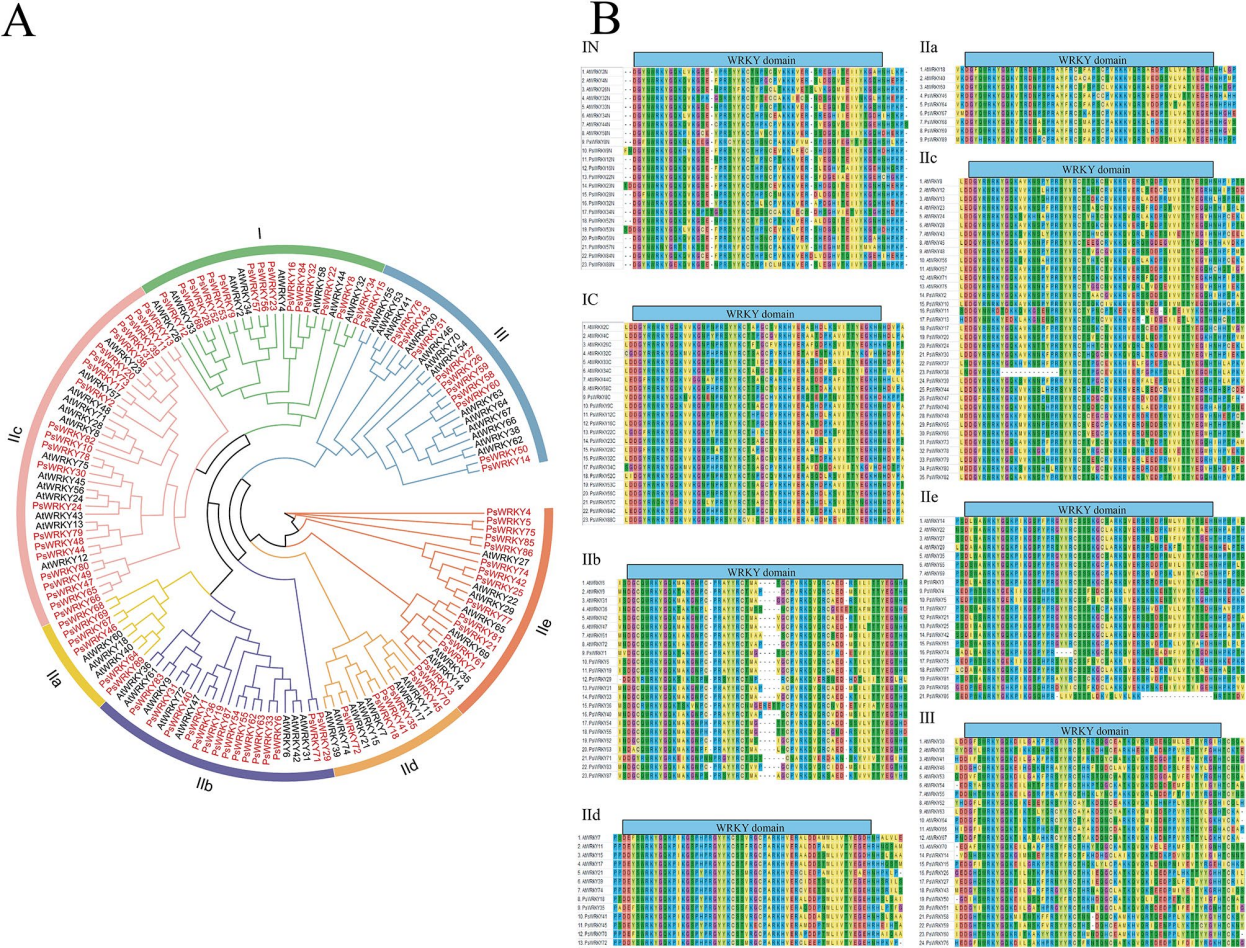
(**A**) Phylogenetic tree of the relationships between the WRKY proteins of pea and *A. thaliana*, I, IIa, IIb, IIc, IId, IIe, and III represent the different subfamilies. (**B**) Multiple sequence alignment of the WRKY domains of seven subfamilies of the pea and *A. thaliana*, using 60 amino acids on either side of the structural domain. “N” and “C” represent the N-terminal and C-terminal WRKY domains of a specific WRKY protein

Based on the amino acid sequences of the 89 identified PsWRKY and the 60 reported AtWRKY proteins, as well as on the classification of *A. thaliana* WRKYs, a phylogenetic tree was constructed using the TBtools software (Bootstrap value of 1,000) (Fig. 1A), and the 89 PsWRKY proteins were classified into seven subfamilies (I, IIa, IIb, IIc, IId, IIe, and III). Among them, subfamily IIc contained the most PsWRKY members (22), followed by subfamilies I and IIb (15), IIe (14), III (11), and IIa and IId (6). The evolutionary tree showed that multiple PsWRKYs are tightly clustered with AtWRKY, indicating that these proteins have high homology and similar gene functions.

To further investigate the evolutionary relationship of the structural domains of the PsWRKY proteins in the seven subfamilies, the amino acid sequences of the PsWRKY and AtWRKY proteins were subjected to multiple sequence comparisons, and 60 amino acid sequences before and after those that contained the WRKY structural domains were selected for analysis (Fig. 1B). Subfamily I contained two WRKY structural domains, while subfamilies IIa, IIb, IIc, IId, IIe, and III all contained only one. As shown in Fig. 1B, the sequences in the WRKY structural domain were highly conserved, and 72 PsWRKY proteins were found to have the highly conserved sequence WRKYGQK; one PsWRKY protein showed loss of the structural domain (PsWRKY38), whereas other PsWRKY proteins (PsWRKY4, PsWRKY5, PsWRKY11, PsWRKY13, PsWRKY47, PsWRKY49, PsWRKY57, PsWRKY65, PsWRKY66, PsWRKY71, PsWRKY75, PsWRKY78, PsWRKY80, PsWRKY85, PsWRKY86, and PsWRKY88) varied only by individual amino acids. Interestingly, almost all PsWRKY in IIb has an amino acid stretch missing, which needs to be explored further.

### Gene structures, conserved motifs, and cis-acting element analysis of the *PsWRKY* genes

**Fig. 2:**
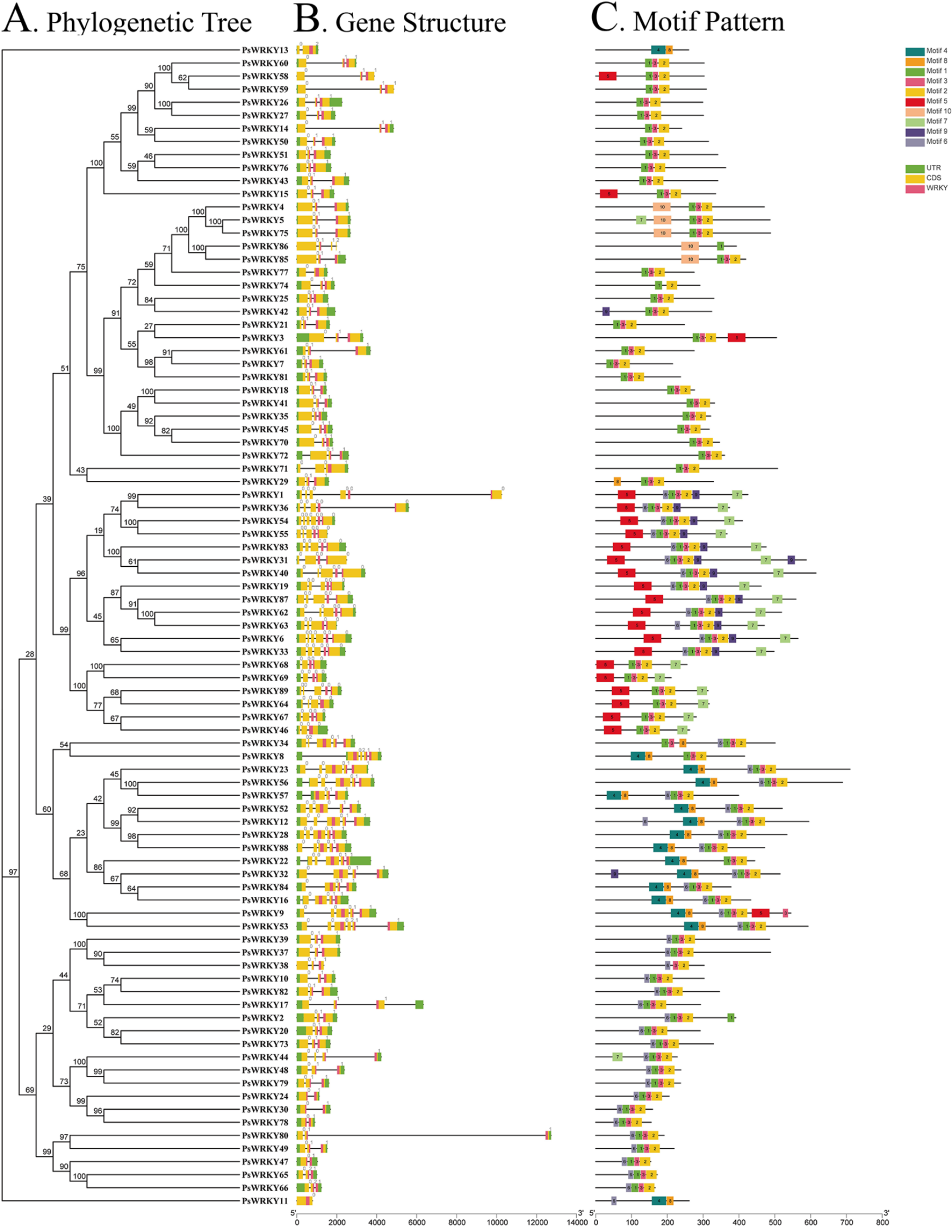
Phylogenetic tree, gene structure, and motif pattern of PsWRKY proteins. (**A**) The phylogenetic tree was constructed using the full-length sequences of PsWRKY proteins with 1000 replicates on each node. (**B**) Green rectangles, yellow rectangles, pink rectangles, and black lines indicate UTR (non-coding region), CDS (coding sequence or exons), WRKY domain, and introns, respectively. (**C**) The amino acid motifs (numbered 1–10) in PsWRKY proteins are displayed in ten colored boxes, and black lines indicate amino acid length

To understand the structural composition of *PsWRKY* genes, a structural map was constructed based on the pea genome sequence, including the untranslated region (UTR), coding sequence (CDS), WRKY structural domains, and introns (Fig. 2A and B). Comparison of the number and location of exons and introns revealed that all 89 *PsWRKY* genes contained different numbers of exons, all containing WRKY structural domains, and that 85 *PsWRKY* genes contained UTRs, except *PsWRKY11*, *PsWRKY38*, *PsWRKY58*, and *PsWRKY86*. Except for one (*PsWRKY11*), the remaining 88 *PsWRKY* genes contained introns, of which 45 (50.56%) contained two introns, 18 (20.22%) contained four, 13 (14.61%) contained three, and 6 (5.62%) contained one or five.

To further investigate the diversity of changes in the PsWRKY family during evolution, the conserved motifs of the 89 PsWRKY proteins were analyzed using MEME online software, and 10 different conserved motifs (named motifs 1–10) were identified (Fig. 2A and C, Table S2). Among them, PsWRKY11 and PsWRKY13 did not contain motifs 1, 3, and 2, PsWRKY86 did not contain motifs 3 and 2, and all other PsWRKY proteins contained motifs 1, 3, and 2, and the ordering of the three motifs was consistently 1, 3, and 2. We also found that motif 5 was mostly distributed among subfamilies IIa and IIb whereas motif 4 was mostly distributed among subfamily I.

From the pea genome sequence, 2000 bp upstream of the start codon (ATG) of the 89 *PsWRKY* genes were selected as promoter sequences, and their cis-acting elements were predicted using the PLANTCARE online website and visualized using TBtools software. Cis-acting elements involved in hormone responses such as ABA, IAA, GA, MeJA, and SA, and cis-acting elements involved in the low-temperature response, light response, and control of circadian rhythms, as well as the cis-regulatory elements necessary for anaerobic induction, were distributed over the promoter sequence. Among the cis-acting elements related to hormones and environmental stress, light-responsive elements and MeJA-responsive elements were the most widely distributed, with 338 and 284 elements, respectively (Table S3, Figure. S1).

### Chromosomal distribution, gene duplication *PsWRKY* genes

**Fig. 3:**
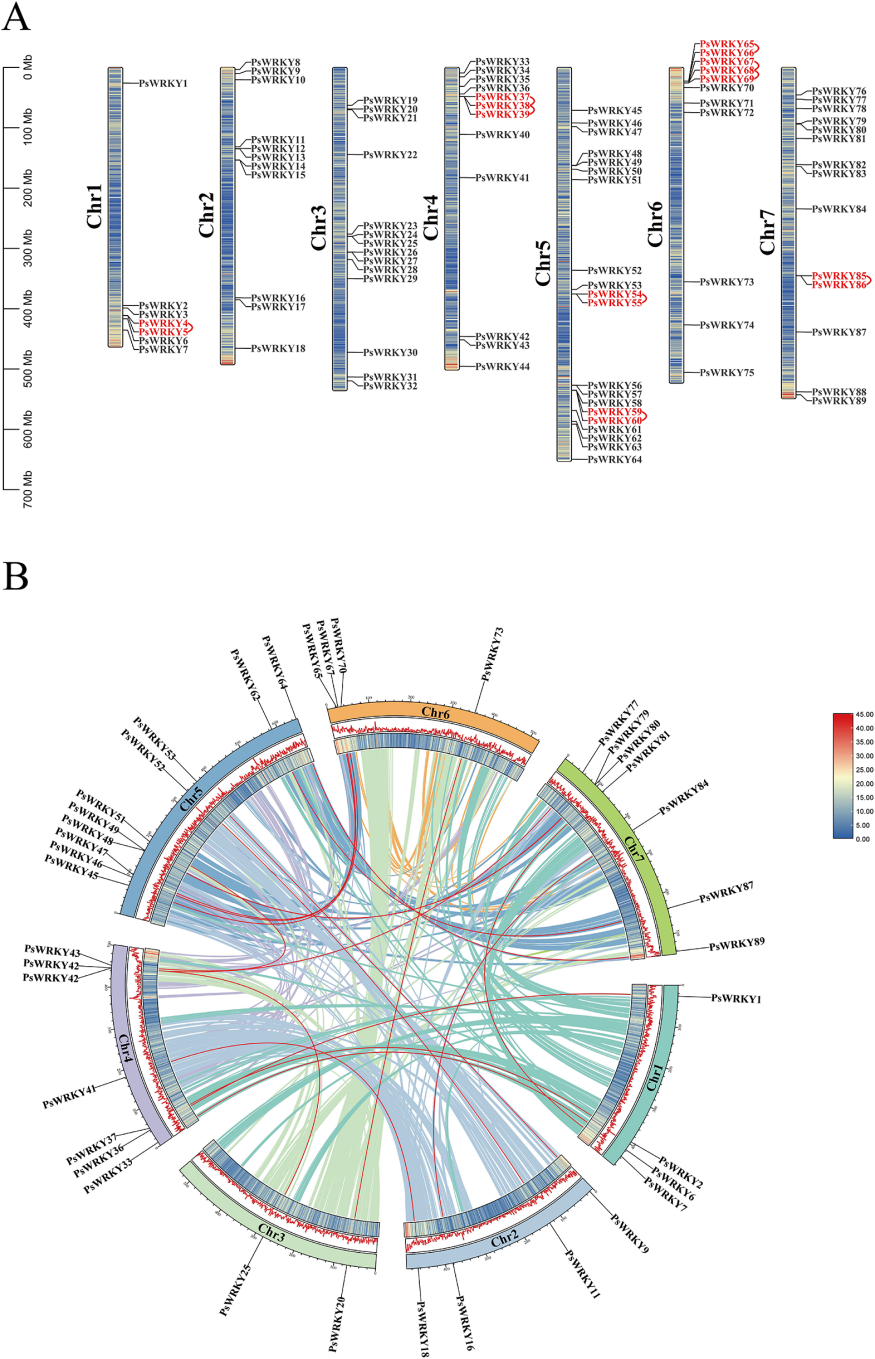
(**A**) Chromosomal location of *PsWRKY*s. The colored rectangular bars represent the chromosomes of pea; Chr 1–7 represents each corresponding chromosome; red fonts represent gene tandem repeats, and the 0–700 Mb scale represents chromosome length. (**B**) Chromosome distribution and gene duplication relationship of *PsWRKYs*. Colored lines indicate the collinear regions in the genomes of pea, and red lines indicate duplicated *WRKY* gene pairs. The chromosome number is shown inside each chromosome

The 89 *PsWRKY* genes were unevenly distributed across the seven chromosomes (Fig. 3A), and genes of the same subfamily were randomly distributed across the chromosomes. Among them, Chr1 contained seven genes (*PsWRKY1*–*PsWRKY7*, 7.87%), Chr2 contained 11 genes (*PsWRKY8*–*PsWRKY18*, 12.36%), Chr3 contained 14 genes (*PsWRKY19*–*PsWRKY32*, 15.73%), Chr4 contained 12 genes (*PsWRKY33*–*PsWRKY44*, 13.48%), Chr5 contained 20 genes (*PsWRKY45*–*PsWRKY64*, 22.47%), Chr6 contained 11 genes (*PsWRKY65*–*PsWRKY75*, 12.36%), and Chr7 contained 14 genes (*PsWRKY76*–*PsWRKY89*, 15.73%).

A chromosomal region within the 200 kb range containing two or more genes was defined as a tandem duplication event [39]. In this study, we observed nine tandem duplication events involving 16 *PsWRKY* genes on chromosomes 1, 4, 5, 6, and 7 (Fig. 3A, Table S4), with two tandem duplicates each of *PsWRKY38* and *PsWRKY68* (*PsWRKY38* and *PsWRKY37*/*PsWRKY39*, and *PsWRKY68* and *PsWRKY67*/*PsWRKY69*). All genes that undergo tandem duplication events belong to the same subfamily; for example, *PsWRKY4* and *PsWRKY5* belonging to subfamily IIe are tandemly duplicated genes, as are *PsWRKY54* and *PsWRKY55* belonging to subfamily IIb. Segmental duplication analysis of the 89 *PsWRKY* genes by BLASTP and MCScanX identified 38 homologous sites and 19 pairs of segmental duplication events (Fig. 3B, Table S5): *PsWRKY2*/*PsWRKY37*, *PsWRKY8*/*PsWRKY41*, *PsWRKY25*/*PsWRKY42*, *PsWRKY43*/*PsWRKY51*, *PsWRKY11*/*PsWRKY52*, *PsWRKY9*/*PsWRKY53*, *PsWRKY46*/*PsWRKY67*, *PsWRKY45/PsWRKY71*, *PsWRKY47*/*PsWRKY65*, *PsWRKY20*/*PsWRKY73*, *PsWRKY64*/*PsWRKY89*, *PsWRKY48/PsWRKY79*, *PsWRKY62*/*PsWRKY87*, *PsWRKY16*/*PsWRKY84*, *PsWRKY7*/*PsWRKY81*, *PsWRKY42*/*PsWRKY77*, *PsWRKY49*/*PsWRKY80*, *PsWRKY6*/*PsWRKY33*, and *PsWRKY1*/*PsWRKY36*. Among these, four pairs of *PsWRKY* genes belonged to subfamilies IIc, IIb, and IIa, each containing three pairs of *PsWRKY* duplicates; subfamilies I and IIe contained two pairs of duplicates; and subfamilies IId and III each contained one pair of duplicates. The family of *PsWRKY* genes was heterogeneously distributed among the seven linkage groups (LG) of peas, with LG5 containing the most *WRKY* genes (10/38, 26.32%) and LG3 containing the least (2/38, 5.26%).

### Collinear analysis and evolutionary analysis of PsWRKY proteins and WRKY proteins of different plants

**Fig. 4:**
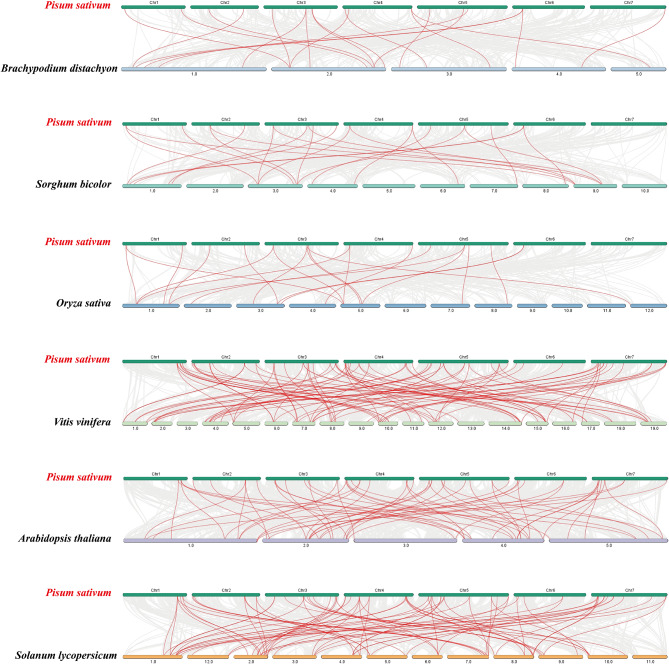
Collinear analyses of *WRKY* genes between pea and six plants (*B. distachyon, O. sativa*, and *S. bicolor, A. thaliana, V. vinifera*, and *S. lycopersicum*. The gray lines between pea and other plants represent collinear blocks in wide regions of the genomes, while red lines show the orthologous relationship of *WRKY* genes

To explore the evolutionary relationship between *PsWRKY* and different species, a collinear maps of pea with three monocotyledons (*Brachypodium distachyon L.*, *O. sativa*, and *Sorghum bicolor* (L.) Moench) and three dicotyledons (*A. thaliana*, *Vitis vinifera L.*, and *Solanum lycopersicum L.*) were constructed (Fig. 4, Table S6). The *PsWRKY* gene family was found to have strong collinear relationship with *WRKY* genes of dicotyledonous plants, with the highest collinear relationship in *V. vinifera.* (90 pairs), followed by *S. lycopersicum* (79 pairs) and *(A) thaliana* (60 pairs). The *PsWRKY* genes shared a few genes with the three monocotyledons, including 23 pairs of genes with *S. bicolor*, 21 with *O. sativa*, and 20 with *(B) distachyon*. *PsWRKY4*, *PsWRKY5*, *PsWRKY*15, *PsWRKY38*, *PsWRKY39*, *PsWRKY50*, *PsWRKY54*, *PsWRKY55*, *PsWRKY57*, *PsWRKY58*, *PsWRKY59*, *PsWRKY60*, *PsWRKY63*, *PsWRKY66*, *PsWRKY68*, *PsWRKY69*, *PsWRKY71*, *PsWRKY74*, *PsWRKY75*, *PsWRKY85*, and *PsWRKY86* did not have co-localized genes with any of the six species, suggesting that these genes may have formed after plant differentiation.

To further explore the affinities of WRKY proteins in pea and the six other plants, a rootless NJ tree was constructed based on the amino acid sequences of the 89 identified PsWRKY proteins and the amino acid sequences of the WRKYs of the six plants (Figure S2, Table S7). Most pea WRKY tended to be on the same branch in *V. vinifera* and *S. lycopersicum*, showing that their relationship with PsWRKY proteins may be closer, compared with the other four plants. The motifs contained in each subfamily differed little (Figure S2, Table S8); for example, subfamily IIa contained motifs 1-3-4-2-10, and subfamily I contained motifs 5-1-3-4-2, suggesting that these proteins have similar structures and functions.

**Fig. 5:**
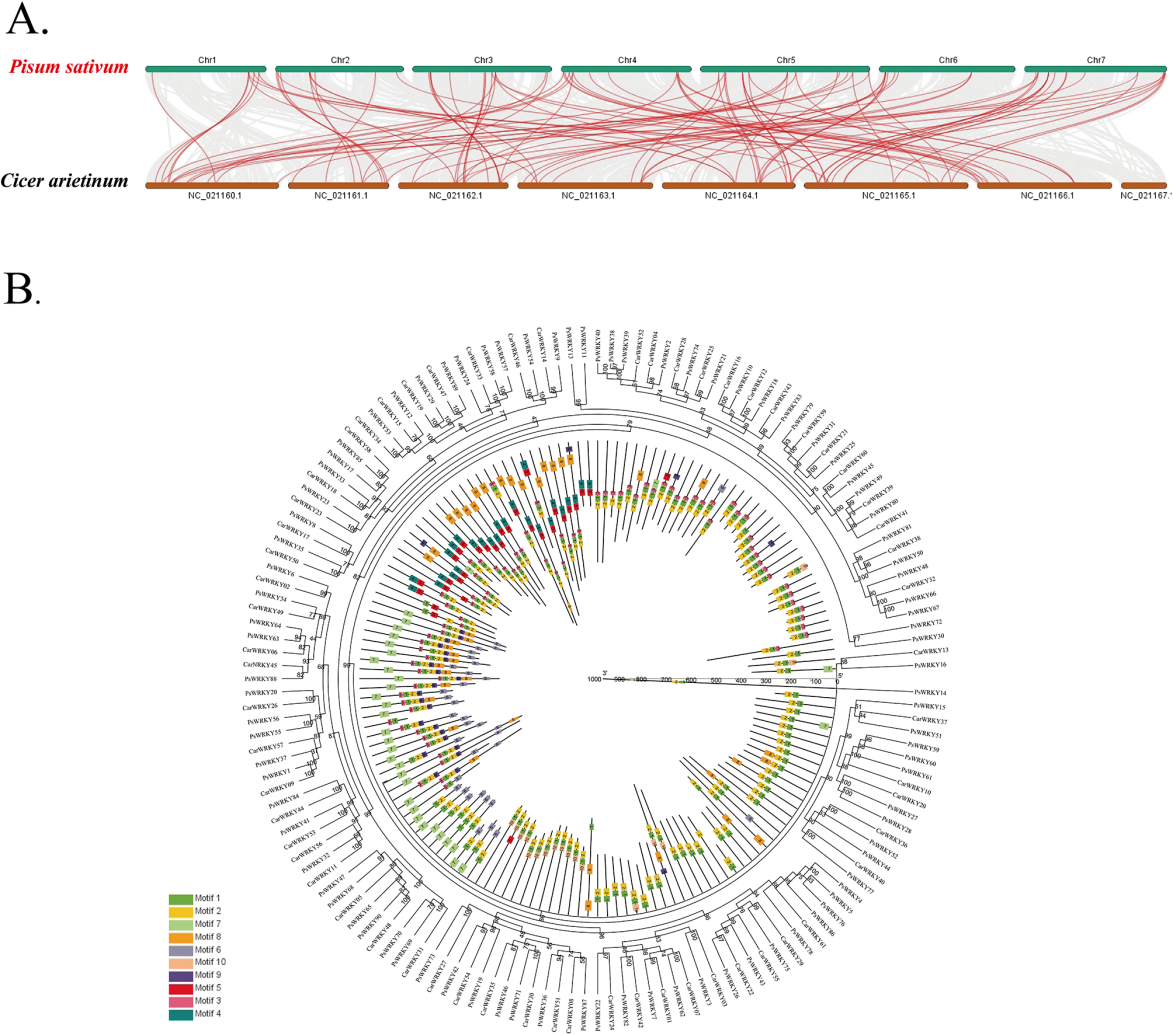
(**A**) Collinear analyses of WRKY genes between pea and chickpea the gray lines between pea and chickpea represent collinear blocks in wide regions of the genomes, while red lines show the orthologous relationship of WRKY genes. (**B**) Phylogenetic relationship and motif pattern of WRKY proteins among pea and chickpea. The colored legends represent the amino acids motifs (numbered 1–10), the outer part of the circle represents the phylogenetic tree of the WRKY proteins of the two plants, and the inner part of the circle represents protein length, conserved motifs, and composition

To investigate the homology of pea WRKYs with other legume WRKYs, we constructed a collinear map and a phylogenetic relationship and motif pattern between pea and chickpea (*Cicer arietinum L.*) (Fig. 5, Table S6, S7, S8), the results showed that pea WRKY has high homology with chickpea WRKY.

### Expression patterns of *PsWRKYs* in different plant tissues and fruit development

**Fig. 6:**
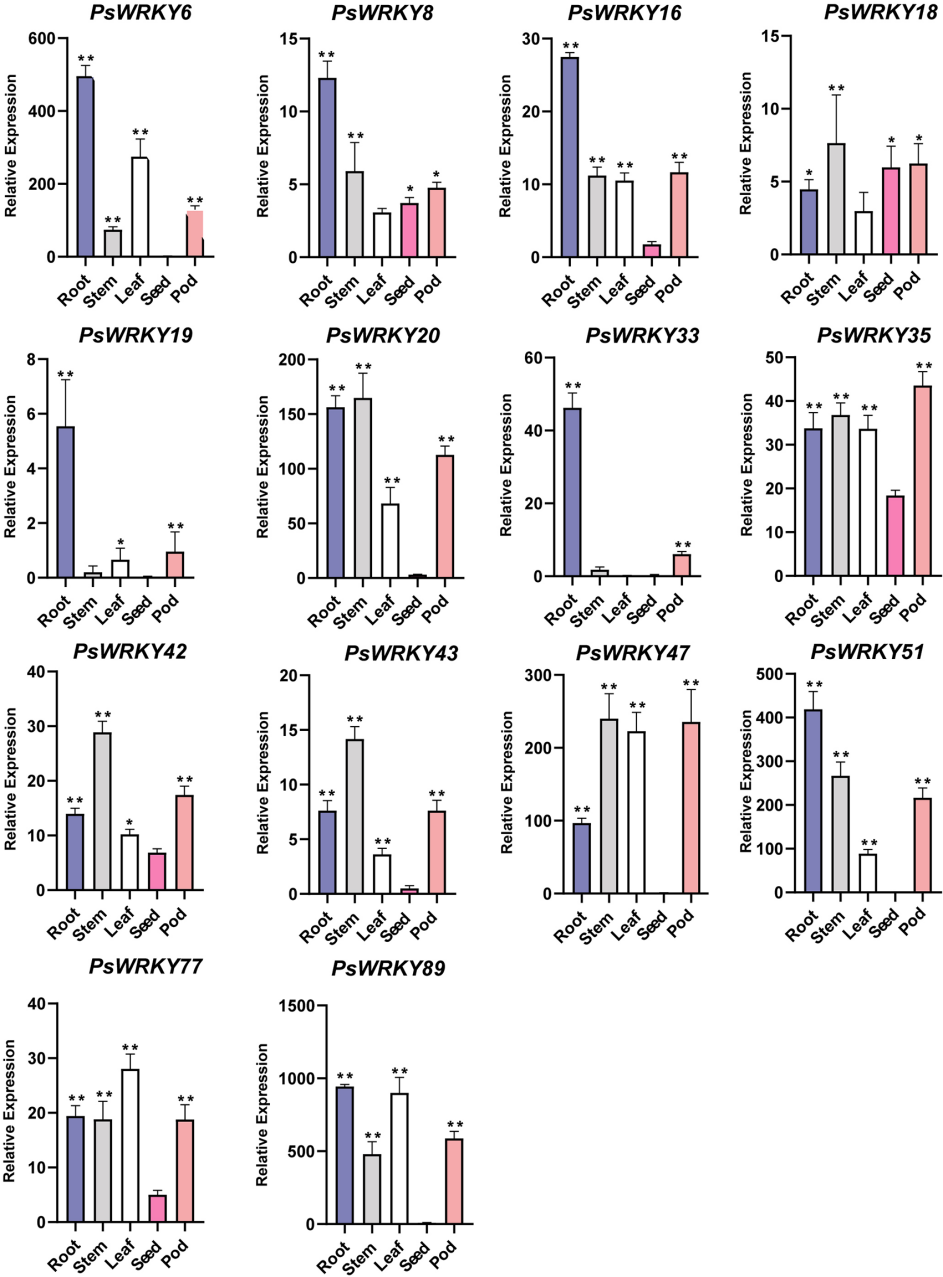
Expression patterns of 14 *PsWRKY* genes in five tissues (roots, stems, leaves, seeds, and pods) of pea at mid-irrigation. The * indicates significant differences by *t*-test (*p < 0.05, **p < 0.01)

**Fig. 7:**
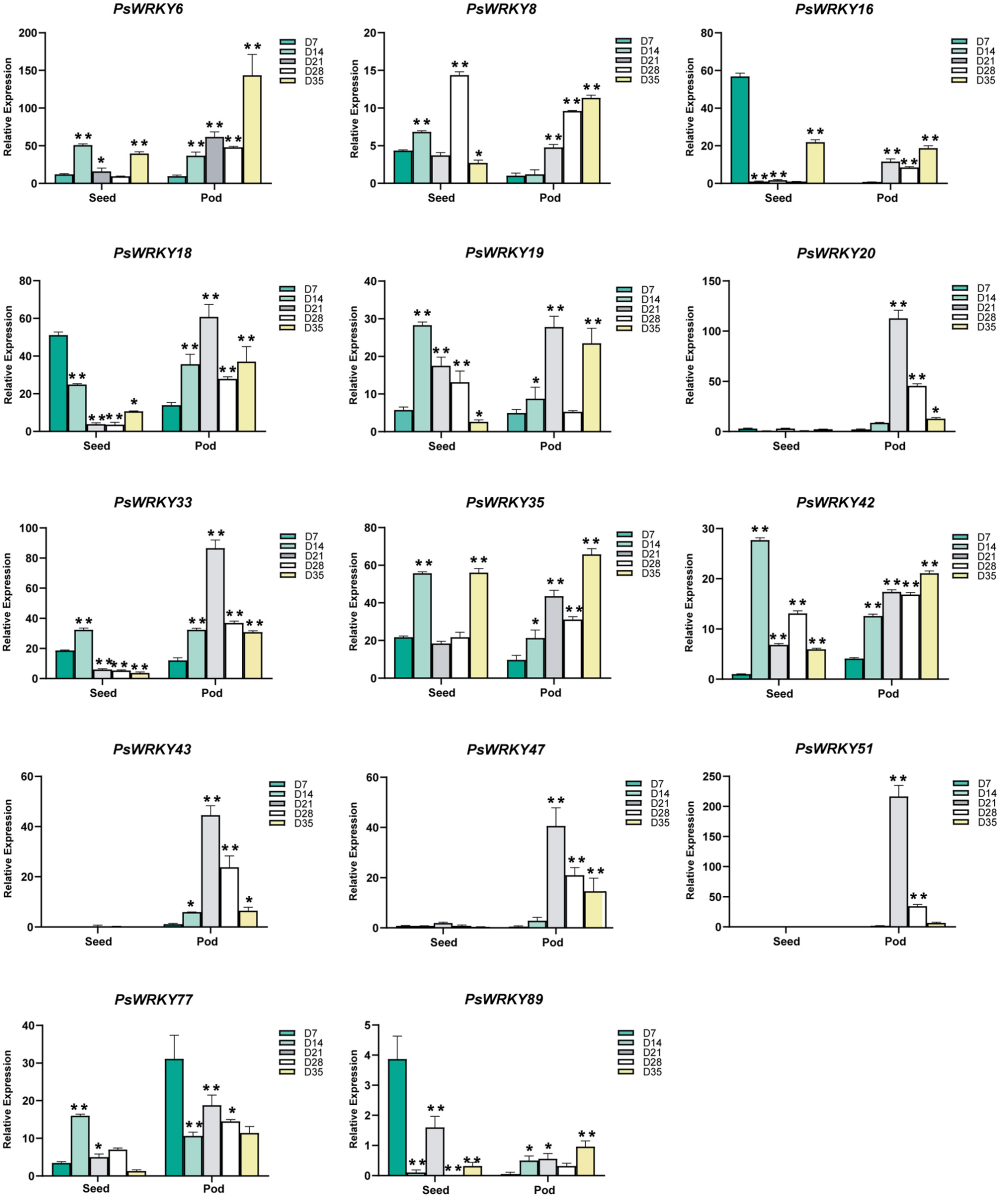
Expression patterns of 14 *PsWRKY* genes in seed and pod at different times of pea grain filling. The * indicates significant differences by *t*-test (*p < 0.05, **p < 0.01)

To further analyze the possible role of *PsWRKY* genes in pea growth and development, 14 *PsWRKY* genes from different subfamilies were selected and analyzed for their relative expression levels in five tissues (roots, stems, leaves, seeds, and pods) at the mid-irrigation stage of pea seed (Fig. 6) using qRT-PCR. Based on the experimental results, we found that most of the *PsWRKY* genes were expressed at high relative levels in roots (*PsWRKY6*, *PsWRKY8*, *PsWRKY16*, *PsWRKY19*, *PsWRKY33*, *PsWRKY51*, *PsWRKY89*), and some genes were expressed in stems (*PsWRKY18*, *PsWRKY20*, *PsWRKY43*, *PsWRKY47*, *PsWRKY53*) leaves (*PsWRKY56*), and pods (*PsWRKY35*), but the overall expression level of *PsWRKY* genes in seeds was low. Pea seeds are an excellent source of protein, dietary fiber, and mineral nutrients, some *PsWRKY* genes may regulate the fruit development of pea, thus affecting its nutritional composition and development rate. so, we analyzed the relative expression levels of the 14 *PsWRKY* genes in seeds and pods at different times of pea seed filling (Fig. 7) and found that most of the *PsWRKY* genes had high relative expression levels in pods during seed and pod development (*PsWRKY6*, *PsWRKY18*, *PsWRKY20*, *PsWRKY33*, *PsWRKY35*, *PsWRKY43*, *PsWRKY47*, *PsWRKY51*, *PsWRKY77*), and some *PsWRKY* genes had high relative expression in seeds (*PsWRKY8*, *PsWRKY16*, *PsWRKY19*, *PsWRKY42*, *PsWRKY89*). Although the expression patterns of each *PsWRKY* gene were not identical in different tissues and at different times in the seeds and pods, we found similarities in the relative expression levels of some genes belonging to uniform subfamilies. For example, among the different tissue expression patterns, both *PsWRKY8* and *PsWRKY16*, which belong to subfamily Ic, had the highest relative expression levels in the roots, and both *PsWRKY20* and *PsWRKY47*, which belong to subfamily IIc, had higher relative expression levels in the stems and the lowest in the seeds. Among the expression patterns in seeds and pods at different times, the relative expression levels of *PsWRKY19* and *PsWRKY33*, which belong to subfamily IIb, were highest at day 14 in both seeds and pods. The relative expression levels of *PsWRKY20* and *PsWRKY47*, which belong to subfamily IIc, were extremely low in seeds, whereas in pods, they both reached their highest expression levels at day 21.

### Expression patterns of *PsWRKY* genes in response to hormone treatments

**Fig. 8:**
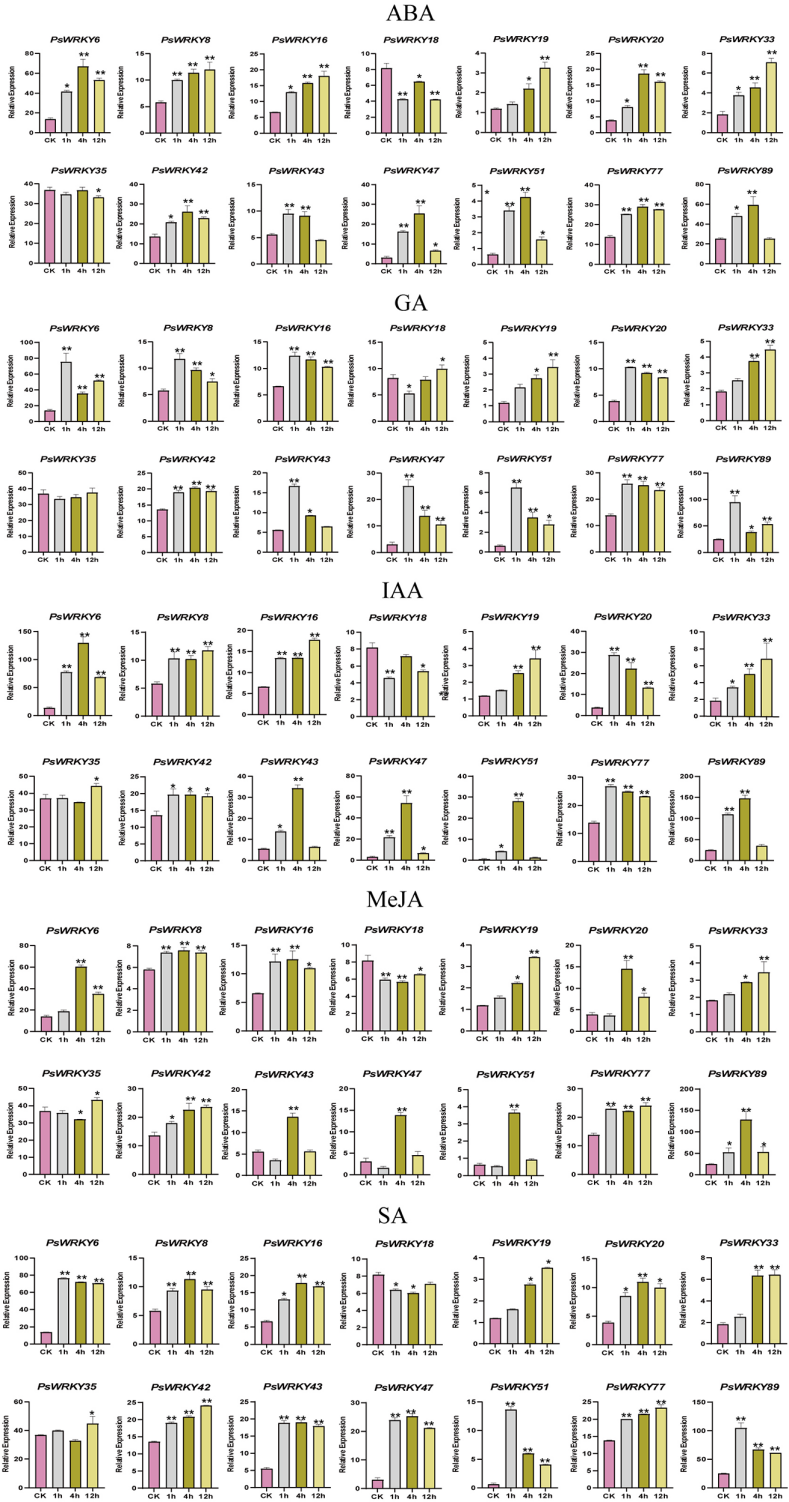
Expression patterns of 14 *PsWRKY* genes in pea seedlings after 5 hormone treatments. The * indicates significant differences by *t*-test (*p < 0.05, **p < 0.01)

Cis-acting element analysis of the 89 *PsWRKY* genes revealed the presence of ABA, MeJA, GA, SA, and IAA hormone-responsive elements, and it has also been reported that *WRKY* genes regulate hormone-mediated signaling pathways, suggesting that *PsWRKY* genes may be responsive to MeJA, GA, SA, and IAA, which are jointly involved in the regulation of pea growth and development. To further investigate the role of *PsWRKY* genes in the response mechanism of the five hormones, pea seedlings were treated with each of the hormones and sampled at 0, 1, 4, and 12 h post-treatment, with 0 as the control, and the expression patterns of the 14 *PsWRKY* genes belonging to seven different subfamilies were analyzed using qRT-PCR (Fig. 8). The relative expression of different genes showed significant differences under different hormone treatments, indicating that all these genes could respond to treatment with these five hormones. Although the expression patterns of each gene were not identical, several similarities were observed. Most of the genes showed an increasing and then decreasing trend after five hormone treatments, such as *PsWRKY6*, *PsWRKY20*, *PsWRKY42*, *PsWRKY43*, *PsWRKY47*, *PsWRKY51*, *PsWRKY77*, and *PsWRKY89* under ABA treatment; *PsWRKY8* and *PsWRKY16* under GA treatment; *PsWRKY20*, *PsWRKY42*, *PsWRKY43*, *PsWRKY47*, *PsWRKY51*, *PsWRKY77*, *PsWRKY6*, *PsWRKY20*, *PsWRKY43*, *PsWRKY47*, *PsWRKY51*, and *PsWRKY89* under IAA treatment; *PsWRKY6* under MeJA treatment; and *PsWRKY8*, *PsWRKY16*, *PsWRKY89*; *PsWRKY6*, *PsWRKY8*, *PsWRKY16*, *PsWRKY20*, *PsWRKY43*, *PsWRKY47*, *PsWRKY51*, and *PsWRKY89* under SA treatment. Some genes showed a continuous increase after hormone treatment, such as *PsWRKY8*, *PsWRKY16*, *PsWRKY19*, and *PsWRKY33* under ABA treatment; *PsWRKY19* and *PsWRKY33* under GA treatment; *PsWRKY8*, *PsWRKY16*, *PsWRKY19*, and *PsWRKY33* under IAA treatment; and *PsWRKY19*, *PsWRKY33*, *PsWRKY42*, and *PsWRKY77* under SA treatment. In most cases, genes belonging to the same subfamily had similar expression patterns.

## Discussion

WRKY TFs are a class of DNA-binding proteins primarily found in plants that respond to a wide range of biotic and abiotic stressors. In this study, we used various bioinformatics methods to identify the whole pea genome, obtained 89 members of the *PsWRKY* gene family, and analyzed their physicochemical properties such as MW and theoretical pI (Table S1). There were significant differences among the 89 PsWRKY proteins, with amino acid lengths ranging from 154 (PsWRKY47) to 710 aa (PsWRKY23) and MW ranging from 17.68 (PsWRKY47) to 79.12 kD (PsWRKY23). This suggests that plants have diverged inconsistently over time as their environment has changed throughout evolution, which, in turn, has led to differences in structure between species. The 89 PsWRKY proteins had a pI ranging from 4.94 (PsWRKY87) to 9.97 (PsWRKY35) and most had pI < 7, indicating that this gene family tends to be enriched in acidic amino acids. This is consistent with studies related to dicotyledons [1] and contrary to those related to monocotyledons [40], which suggests that monocotyledons and dicotyledons diverged during the evolutionary process. From the predicted subcellular localization results of the 89 PsWRKYs, we found that they were localized to the nucleus, cytoplasm, mitochondria, plasma membrane, chloroplast, Golgi apparatus, extracellular and peroxisomes (Table S1); however, the majority of the *PsWRKY* genes were localized to the nucleus (82/89, 92.13%), indicating that the pea *WRKY* gene family plays a dominant role in controlling plant growth and development, mainly within the nucleus. Based on the family classification of *Arabidopsis thaliana* WRKY, the 89 PsWRKY proteins were categorized into three main groups (groups I, II, and III), and group II was further divided into five subgroups (IIa, IIb, IIc, IId, and IIe) (Fig. 1A). This subfamily grouping is consistent with most WRKY family studies [41, 42], suggesting relatively stability during evolution. By further multiple sequence comparison of the amino acid sequences of PsWRKY and AtWRKY (Fig. 1B), group I was found to contain two WRKY domains located at the C-terminal- and N-terminal ends of the sequences, whereas groups II and III contained only one WRKY domain, which is in agreement with the findings of other studies [43]. Among the 89 PsWRKY proteins, most of the WRKY domains were highly conserved and one PsWRKY protein showed a loss of structural domains (PsWRKY38), suggesting that this protein may play a different role from the other proteins. It is noteworthy that almost all genes in subfamily IIb have amino acid stretch missing, which have also been found in other species [44–46], therefore, we hypothesize that the amino acid stretch missing in class IIb is due to some function of subfamily IIb by fragment missing during pea evolution, and the exact reason for this needs to be further investigated. There are differences in conserved sequences among different subfamilies, and WRKY domains are not identical within the same subfamily, which allows PsWRKY proteins to perform different functions during plant growth and development.

Although the physicochemical properties of the Pea WRKY family, such as protein length, MW, and pI, were highly variable, the gene structure and amino acid motifs were relatively conserved (Fig. 2A–C). In this study, the 89 identified *PsWRKY* genes were distributed with abundant CDS and intron regions, and it has been shown that the higher the number of introns, the longer the gene sequences and the higher the recombination frequency among the genes [47]. Conserved motif analysis of PsWRKY proteins revealed 10 motifs, and most of the PsWRKY proteins were distributed with motifs 2 and 3, suggesting that motifs 2 and 3 may be the WRKY motifs of this family. Combined with the gene structure and conserved protein motif analysis of PsWRKY members, we found that members of the same subfamily have similar gene structures and conserved motif distributions among themselves, while different subfamilies have larger differences, suggesting conservation within the same subfamily and similar functions, which is consistent with the results of Li et al. (2022) and Wei et al. (2022) [48, 49].

Gene promoters are DNA sequences located upstream of the coding region of a gene that contains multiple cis-acting elements that are specific binding sites for proteins involved in initiating and regulating transcription [50]. In this study, we predicted the cis-acting elements of the 89 *PsWRKY* genes based on their promoter sequences (Fig. 3, Table S3) and found that the promoter regions contained multiple response elements such as for abiotic stresses and phytohormones, with the light-responsive element and the MeJA-responsive element being more widely and broadly distributed, which also suggests that the pea *WRKY* genes can be involved in plant growth, development, and hormonal signaling pathways.

The number of members of the same gene family varies among different species, which may be due to the occurrence of gene recombination, gene duplication, and segmental duplication during evolution and the natural differentiation of species to produce different family members [51, 52]. In this study, we found that the 89 *PsWRKY* genes were randomly distributed on seven chromosomes of peas (Fig. 4A, Table S4), with varying numbers of genes on each chromosome with the highest number of genes distributed on Chr5, suggesting that each of them has a different expression pattern. Previously, 98 *HsWRKYs* and 103 *HvWRKYs* were identified in the reference genomes of wild and cultivated barley, respectively, and tandem and segmental repeat events were observed in the cultivated barley [53]. In this study, we observed *PsWRKY* gene duplication events (Fig. 4B, Table S5): on chromosomes 1, 4, 5, 6, and 7, we identified nine tandem duplication events involving 16 *PsWRKY* genes, and all genes that underwent tandem duplication events were from the same subfamily, suggesting that members of these families may work together to encode proteins and regulate related biological processes. Nineteen pairs of *PsWRKY* fragment duplication events were identified in the pea *WRKY* family. Additionally, we analyzed the covariance of *WRKYs* between pea and four dicotyledons (*A. thaliana*, *V. vinifera*, *S. lycopersicum*, and *chickpea.*) and three monocotyledons (*B. distachyon, O. sativa*, and *S. bicolor*,). The collinear relationships of *WRKYs* between the family of pea *WRKYs* and dicotyledons were found to be greater, and less with monocotyledons (Fig. 5, Table S6), and the results of the analysis of the evolutionary relationships also revealed that pea *WRKYs* clustered more closely with dicotyledonous plant *WRKYs* (Fig. 6, Table S7), which may be related to the classification of monocotyledonous and dicotyledonous plants produced by angiosperms during long-term natural selection and evolution. Some *PsWRKYs* were not co-localized in any of the six plants, suggesting that these genes may have been formed after pea divergence, which led to the conclusion that members of the pea *WRKY* gene family were able to be somewhat amplified, and it can be conjectured that these increased *PsWRKY* genes played an important role in the evolution of pea. The pea *WRKYs* clustered more closely with those of the dicotyledonous plants, suggesting a closer relationship to the dicotyledonous plant *WRKYs*.

The WRKY TFs family is involved in developmental processes and defense responses such as plant seed germination, pollen development, hormone regulation, and biosynthesis of secondary metabolites [54] and plays an important role throughout the developmental period of the plant; however, *WRKY* genes do not always act directly on the target genes, and the same genes may be responsive to a wide range of stresses or treatments [55]. Some WRKY TFs have been reported to be positive regulators of ABA-mediated stomatal closure, while others are negative regulators of seed germination and also indirectly control flowering [56]. Ectopic expression of *TaWRKY75-A* and *ZmWRKY79* in *Arabidopsis thaliana L.* improves survival under drought stress by regulating ABA biosynthesis [57, 58]. Kalde et al. (2003) found that the expression of *AtWRKY21* was induced by SA [59]. *CrWRKY36*, a homologue of *AtWRKY21*, was upregulated 2 h after MeJA treatment [60]. The *AtWRKY3* gene is involved in both the SA and JA signaling pathways. The *AtWRKY3* gene plays a negative role in the SA signaling pathway, which mediates resistance to biotrophic pathogens and plays a positive role in MeJA-mediated resistance to necrotrophic pathogens [61]. To investigate the potential function of *PsWRKY* genes, we examined the expression of 14 *PsWRKY* genes of seven subfamilies in roots, stems, leaves, seeds, and pods, to explore whether the *PsWRKY* genes function in different tissues of peas. There were significant differences in the expression levels of different genes in various tissues during the same period, with some genes having higher relative expression in pea roots, and others in stems or leaves, this finding is similar to that of Yang et al. that *WRKY* genes belonging to different subfamilies in soybean are expressed to varying degrees in tissues such as roots, leaves, and seeds [19]. Then, to explore the role of *PsWRKYs* during the development of pea fruit, we detected the expression of 14 *PsWRKY* genes in seeds and pods at different periods of pea seed irrigation and found that during the period of fruit development, most of the genes with lower expression in pea seeds and higher expression in pods. This is similar to the findings of Gu et al., according to their experimental results, it is known that during soybean fruit development, the expression of *SoyWRKY15a* is higher in pods than in seeds [62]. The above results led to the conclusion that the *PsWRKY* family is tissue-specific and that the expression of this gene family is relatively low in pea seeds, suggesting they may indirectly affect the development of pea seeds by participating in the response in pea tissues such as roots, stems, leaves, and pods.

The SA and MeJA signaling pathways have been shown to play opposing roles in defense, but synergistic effects between these phytohormones have also been reported [63]. Based on cis-acting element analysis of the promoters of the identified *PsWRKY* genes, hormone-responsive elements were found, and it has also been reported that *WRKY* genes regulate hormone-mediated signaling pathways, thus, we treated 14 *PsWRKY* genes with five hormones (ABA, MeJA, GA, SA, and IAA), and detected the expression of the genes at different times after the treatments by qRT-PCR. The expressions showed different degrees of variation, some showed an increasing and then a decreasing trend, whereas others showed a continuously increasing trend. From these results, it can be concluded that pea *WRKY* genes respond to hormone treatments, and there is a synergistic effect between hormones.

The above results indicate that the expression pattern of the pea *WRKY* gene family is diverse in different tissues, at different times, and under different hormone treatments, suggesting functional diversification of the pea *WRKY* gene family as well as its key roles in the pea tissue development and hormone response.

## Conclusion

In this study, 89 *PsWRKY* genes were systematically identified for the first time from the whole genome of pea and categorized into seven subfamilies (I, IIa, IIb, IIc, IId, IIe, and III), and it was found that the physicochemical properties of the *PsWRKY* family varied significantly, whereas the gene-results and the conserved sequences of the proteins had a high degree of conservatism. The 89 *PsWRKY* genes were randomly distributed across seven chromosomes, with nine pairs of gene tandem duplication events and 19 pairs of fragment duplication events, and the *PsWRKY* genes showed the highest degree of homology to dicotyledonous plants. Additionally, the *PsWRKY* family was tissue-specific during growth and development and responded to phytohormone signals. This study provides a theoretical basis and lays the foundation for future research on the function and mechanism of action of pea *WRKY* genes in plant growth and development.

## Materials and methods

### Genome-wide identification of *WRKY* genes in Pea

First, the Hidden Markov model (HMM) file corresponding to the WRKY structure domain (PF03106) was obtained from the PFAM database (https://www.ebi.ac.uk/Tools/hmmer/search/phmmer). Next, the *A. thaliana* (https://www.arabidopsis.org/) and *O. sativa* (http://rice.uga.edu/) *WRKY* gene sequences were used for comparison and de-redundancy in the pea genome file using BLASTp (score value ≥ 100, e-value ≤ 1e-10) [64] to screen out all possible PsWRKY proteins. Finally, the conserved structural domains were searched using the CD-Search database (https://www.ncbi.nlm.nih.gov/Structure/cdd/cdd.shtml), the SMART tool (http://smart.embl-heidelberg.de/) used to identify *PsWRKY* candidate genes, and the physicochemical properties and subcellular localization of the identified PsWRKY family members predicted using the online websites ProtParam (https://web.expasy.org/protparam/), WoLF PSORT (https://psort.hgc.jp/), and Cell-PLoc (http://www.csbio.sjtu.edu.cn/bioinf/Cell-PLoc/).

## Gene structure, conserved motifs, and cis-acting elements

Multiple sequence comparison analysis of the pea and *A. thaliana* WRKY families was performed using MEGA 11 software based on ClustalW default parameters [65]. A structural map of *PsWRKY* genes was constructed from pea genomic data using TBtools software [64]. Conserved motifs of the PsWRKY protein family were predicted using the online software MEME (https://meme-suite.org/meme/tools/meme), with the maximum conserved motif search value set to 10, and the remaining parameters set to default [66]. Cis-acting elements in the *PsWRKY* gene family promoter sequence (upstream 2000 bp) were predicted using the PlantCare website (http://bioinformatics.psb.ugent.be/webtools/plantcare/html/).

### Chromosomal distribution of *PsWRKY* genes

*PsWRKY* genes were localized to the pea chromosome using Circos [67] based on Pea genomic data. Tandem and segmental duplication events of the *PsWRKY* genes were analyzed using the Multiple Collinearity Scan toolkit X (MCScanX) [68] with default parameters, and the Dual Synteny Plotter was used to analyze tandem duplication events and homology of the *WRKY* genes in pea and seven other species (*B. distachyon*, *O. sativa*, *S. bicolor*, *A. thaliana*, *V. vinifera*, *S. lycopersicum*, *and* chickpea.) [64].

### Phylogenetic evolution and classification of the *PsWRKY* gene family

*B. distachyon*, *O. sativa*, *S. bicolor*, *A. thaliana*, *V. vinifera*, and *S. lycopersicum* were obtained from the UniProt database (https://www.uniprot.org/) and chickpea were obtained from the NCBI (https://www.ncbi.nlm.nih.gov/genome/) and iTAK (http://itak.feilab.net/cgi-bin/itak/index.cgi) [69] (Table S7). The Muscle Wrapper model was used to compare the WRKY amino acid sequences of these seven species and pea, and the developmental evolutionary tree constructed using the IQ-Tree Wrapper program in the TBtools software, with the bootstrap number set to 1000 and other parameters as default. An evolutionary tree between pea and *A. thaliana* was constructed as described above, and the identified *PsWRKYs* were analyzed according to the classification and grouping of *AtWRKYs* in the model plant *A. thaliana*.

## Plant materials and stress treatment

The pea cultivar “ZW6” was bred by the Institute of Animal Husbandry, Chinese Academy of Agricultural Sciences. “ZW6,” which was of uniform size and free of pests and diseases, was cultivated in a greenhouse using soil in Chongxue Building 443, Guizhou University, under the following conditions: 16 h/25°C during the daytime and 8 h/20°C at night [70, 71]. The pods and seeds of peas were taken on days 7, 14, 21, 28, and 35 of post-flowering pod development, and roots stems, and leaves were taken at the same time on day 21, and the tissues were rapidly placed in liquid nitrogen and stored in a -80 °C refrigerator [72]. To further investigate the response of *WRKY* genes to hormones in pea, five hormone treatments (ABA: 100 µmol/L, indole-3-acetic acid (IAA): 100 µmol/L, gibberellin (GA): 100 µmol/L, MeJA: 100 µmol/L, and SA: 100 µmol/L) were applied to treat 21-day-old pea seedlings [70], and samples taken at 0, 1, 4, and 12 h after treatment. Seedling whole plants and samples were quickly placed in liquid nitrogen and stored in a -80 °C refrigerator [1]. Five biological replicates and three technical replicates were used in each experiment, each hormone treatment experiment was repeated three times.

### Total RNA extraction, reverse transcription, and qRT-PCR analysis

Pea samples (0.1 g) were thoroughly ground in liquid nitrogen and total RNA was extracted using the E.Z.N.A. Plant RNA Kit (Omega Bio-Tek, Inc., USA). Extracted RNA integrity was determined using 1% agarose gel electrophoresis, and RNA purity and concentration were measured using a spectrophotometer (Beijing Kaiao Technology Development Co., Ltd., China). The RNA was reverse transcribed to cDNA and the cDNA was diluted using HiScript II Q RT SuperMix for qPCR Kit (Vazyme Biotech Co., Ltd, China) in a 20 µL reaction system.

Specific primers for qRT-PCR were designed using the Primer Premier software (version 5.0; Premier, Canada), with *PsACTIN* as the internal reference gene (Table S9). The ChamQ Universal SYBR qPCR Master Mix Kit (Vazyme Biotech Co., Ltd, China) was used to generate a 20 µL reaction system: 1.0 µL of template cDNA, 10.0 µL of 2 × SYBR mixture, 0.4 µL of each forward and reverse primer, and ddH2O. qPCR was performed using the CFX96 real-time system (Bio-Rad, Hercules, California, USA). The relative expression levels of the target and internal reference genes were calculated using the 2^−∆∆Ct^ formula [73].

### Statistical analysis

Microsoft Excel 2010 was used for data entry and statistical analyses, and GraphPad Prism 7.0 (GraphPad Software, LLC, San Diego, California, USA) was used to draw the bar graphs.

### Electronic supplementary material

Below is the link to the electronic supplementary material.

Supplementary Material 1

Supplementary Material 2

Supplementary Material 3

Supplementary Material 4

Supplementary Material 5

Supplementary Material 6

Supplementary Material 7

Supplementary Material 8

Supplementary Material 9

Supplementary Material 10

Supplementary Material 11

## Data Availability

The Complete pea (ZW6) genome sequence information was obtained from the data published by Yang et al., (2022). The datasets supporting the conclusions of this study are included in the article and its additional files. Further inquiries can be directed to the corresponding author.
