# Correction: Genome-wide identification, structural characterization and gene expression analysis of the WRKY transcription factor family in pea (*Pisum sativum* L.)

**DOI:** 10.1186/s12870-024-04850-x

**Published:** 2024-02-28

**Authors:** Ruiqi Xiong, Zhonghua Peng, Hui Zhou, Guoxing Xue, Ailing He, Xin Yao, Wenfeng Weng, Weijiao Wu, Chao Ma, Qing Bai, Jingjun Ruan

**Affiliations:** 1 College of Agriculture, Guizhou University, Huaxi District, 550025 Guiyang, Guizhou Province PR China; 2 Sichuan Province Seed Station, 610041 Chengdu, Sichuan China

**Correction: BMC Plant Biol 24, 113 (2024)**

10.1186/s12870-024-04774-6

Following publication of the original article [1], author would like to correct the funding statement in the Funding section. The updated funding is given below and the changes have been highlighted in **bold typeface**.

Original Funding

Not applicable.

Updated Funding

**This work was supported by the Key Laboratory of Molecular Breeding for Grain and Oil Crops in Guizhou Province (Qiankehezhongyindi (2023) 008), the Key Laboratory of Functional Agriculture of Guizhou Provincial Higher Education Institutions (Qianjiaoji (2023) 007) and the National Science Foundation of China (32,161,143,005, 32,160,669, 32,372,051).**

The correction does not affect the overall result or conclusion of the article. The original article has been corrected.

## References

[1] Xiong R, Peng Z, Zhou H et al. Genome-wide identification, structural characterization and gene expression analysis of the WRKY transcription factor family in pea (*Pisum sativum L*). BMC Plant Biol. 24, 113 (2024). 10.1186/s12870-024-04774-6. 10.1186/s12870-024-04774-6 PMC10870581 38365619

