# Supplementary figures and images for: Genome-wide identification, structural characterization and gene expression analysis of the WRKY transcription factor family in pea (Pisum sativum L.)
Source: BMC Plant Biol. 2024 Feb 16;24:113. doi: 10.1186/s12870-024-04774-6 (PMC10870581; doi:10.1186/s12870-024-04774-6)

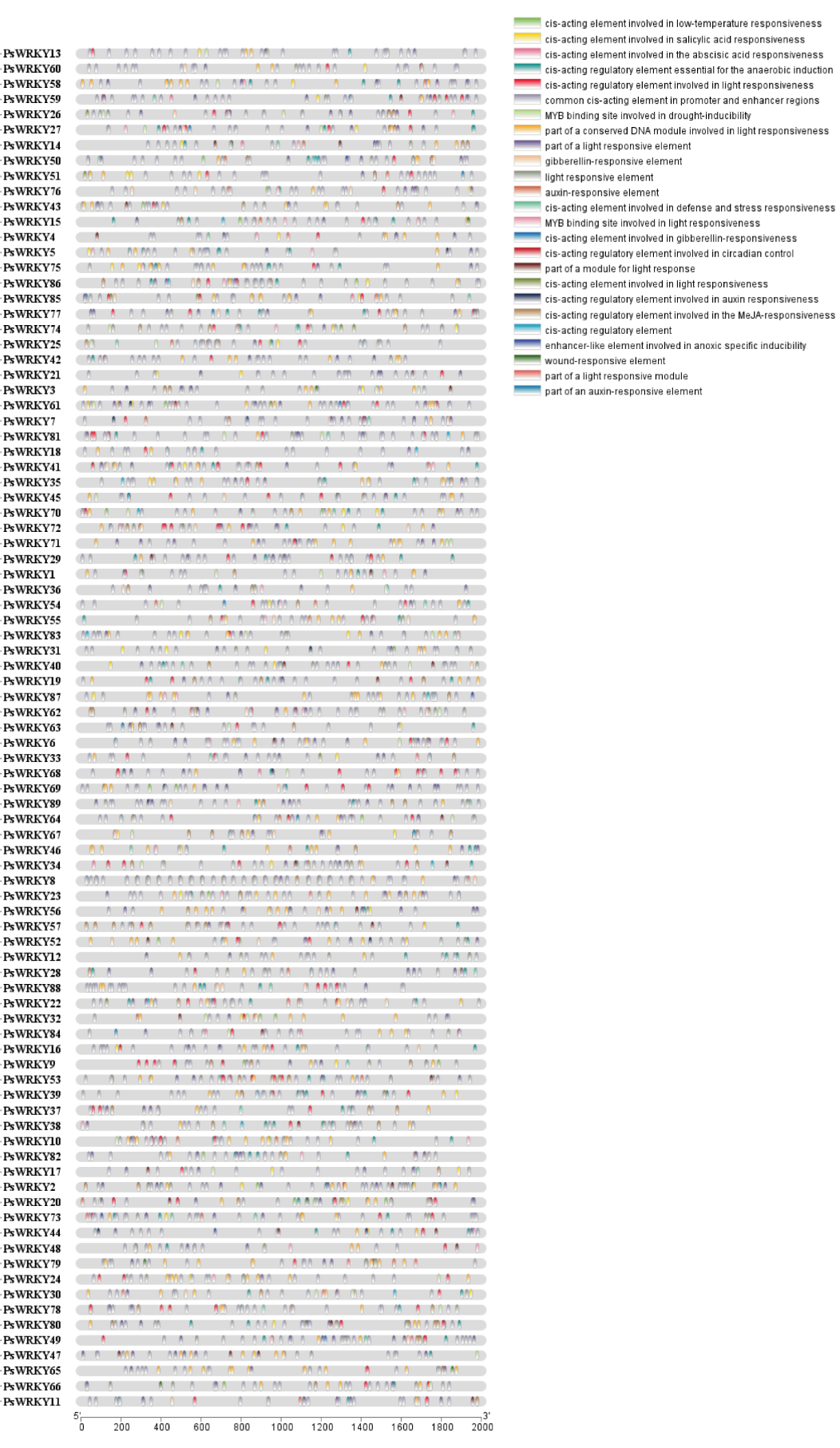


**Figure. S1 The cis-acting element of the promoter region (upstream 2000 bp) of *PsWRKYs***

Supplement: Supplementary file 10 — Supplementary Material 10 [file 12870_2024_4774_MOESM10_ESM.docx]
