# Supplementary material for: Genome-wide identification, structural characterization and gene expression analysis of the WRKY transcription factor family in pea (Pisum sativum L.)
Source: BMC Plant Biol. 2024 Feb 16;24:113. doi: 10.1186/s12870-024-04774-6 (PMC10870581; doi:10.1186/s12870-024-04774-6)
Supplement: Supplementary file 11 — Supplementary Material 11 [file 12870_2024_4774_MOESM11_ESM.docx]

**Figure. S2** Phylogenetic relationship and motif pattern of WRKY proteins among pea and six plants. (B. distachyon, O. sativa, and S. bicolor, A. thaliana, V. vinifera, and S. lycopersicum. ). The colored legends represent the amino acids motifs (numbered 1–10), the outer part of the circle represents the phylogenetic tree of the WRKY proteins of the seven plants, and the inner part of the circle represents protein length, conserved motifs, and composition.
